# Caring for Pregnant Women with Opioid Use Disorder in the USA: Expanding and Improving Treatment

**DOI:** 10.1007/s13669-016-0168-9

**Published:** 2016-07-01

**Authors:** Kelley A. Saia, Davida Schiff, Elisha M. Wachman, Pooja Mehta, Annmarie Vilkins, Michelle Sia, Jordana Price, Tirah Samura, Justin DeAngelis, Clark V. Jackson, Sawyer F. Emmer, Daniel Shaw, Sarah Bagley

**Affiliations:** 1 Department of Obstetrics and Gynecology, Boston Medical Center, 85 East Concord Street, 6th Floor, Boston, MA 02118 USA; 2 Department of Pediatrics, Boston Medical Center, Boston, USA; 3 Department of Family Medicine, Boston Medical Center, Boston, USA; 4 Boston University School of Public Health, Boston, USA; 5 Boston of University School of Medicine, Boston, USA; 6 Department of Psychiatry, Boston Medical Center, Boston, USA; 7 Department of Internal Medicine, Boston Medical Center, Boston, USA

**Keywords:** Pregnancy, Opioid use disorder, Opioid agonist treatment, Naltrexone, Trauma-informed care, Mental health disorder, Breastfeeding

## Abstract

**Purpose of the Review:**

Opioid use disorder in the USA is rising at an alarming rate, particularly among women of childbearing age. Pregnant women with opioid use disorder face numerous barriers to care, including limited access to treatment, stigma, and fear of legal consequences. This review of opioid use disorder in pregnancy is designed to assist health care providers caring for pregnant and postpartum women with the goal of expanding evidence-based treatment practices for this vulnerable population.

**Recent Findings:**

We review current literature on opioid use disorder among US women, existing legislation surrounding substance use in pregnancy, and available treatment options for pregnant women with opioid use disorder. Opioid agonist treatment (OAT) remains the standard of care for treating opioid use disorder in pregnancy. Medically assisted opioid withdrawal (“detoxification”) is not recommended in pregnancy and is associated with high maternal relapse rates. Extended release naltrexone may confer benefit for carefully selected patients. Histories of trauma and mental health disorders are prevalent in this population; and best practice recommendations incorporate gender-specific, trauma-informed, mental health services. Breastfeeding with OAT is safe and beneficial for the mother-infant dyad.

**Summary:**

Further research investigating options of OAT and the efficacy of opioid antagonists in pregnancy is needed. The US health care system can adapt to provide quality care for these mother-infant dyads by expanding comprehensive treatment services and improving access to care.

## Introduction

Over the last decade, prescription opioid misuse and illicit heroin use have skyrocketed, and overdose deaths have quadrupled [1]. From 2002 to 2013, the largest increase in heroin use was among women [2]. A 2014 SAMHSA report showed female treatment admissions for opioid pain relievers as the primary substance of abuse outnumber male admissions in all age categories [3]. The rate of opioid use during pregnancy is approximately 5.6 per 1000 live births [4], with one study reporting greater than 85 % of pregnancies in women with opioid use disorder were unintended [5]. Given the high rates of opioid use among US women of childbearing age, and high incidence of unintended pregnancy in those with opioid use disorder, obstetric providers will need to be prepared to care for this growing demographic.

Despite the increasing acceptance of substance use disorder as a chronic illness, only 11 % of the 24 million Americans with substance use disorder receive treatment [6], and stigma remains a significant barrier to treatment. For pregnant women, this is especially potent. Barriers to care include lack of access to gender-specific care, limited child-care availability at treatment facilities [7], few providers with obstetrics and addiction treatment expertise [8], increased social stigma, and fear of criminal or child welfare consequences. Only 19 states have funded drug treatment programs specifically designed for pregnant women, and 12 states provide pregnant women with priority access to treatment programs.

State policies regarding the response to and reporting of substance use disorder in pregnancy vary wildly. Recently in some states, policymakers have debated legislation criminalizing pregnant women with substance use disorders. In 2014, Tennessee became the first state to pass a law criminalizing illicit drug use during pregnancy.^1^ Eighteen states require health care professionals to report suspected prenatal use of illicit substances, four require health care professionals to test for prenatal drug exposure if substance use disorders are suspected, and 18 states consider substance abuse during pregnancy to be child abuse under civil child-welfare status [9].

Low-income women and women of color are at higher risk for barriers to appropriate care of substance use disorders during pregnancy, in part explaining the poor perinatal outcomes associated with this population [10]. One study found a clear association between little or no prenatal care and opioid use, with a cohort of postpartum patients reporting external locus of control, fear of being reported to the police, and disbelief in the efficacy of care as factors [11].

In the context of rising opioid deaths and increased attention from policymakers, the Department of Health and Human Services has suggested three strategies to reduce deaths: safe prescribing, expansion of medication treatment, and expansion of naloxone access.

In the following review, we will address pharmacologic treatment options for pregnant women with opioid use disorder, with special consideration of mood disorders, trauma history, and breastfeeding.

## Should Pregnant Women with Opioid Use Disorder Undergo “Detoxification”?

Opioid withdrawal (detoxification) in pregnancy is complex and has both risks associated with withdrawal and of relapse for the mother and the fetus. Current practice recommendations are to avoid opioid withdrawal (detoxification) as the benefits of opioid agonist treatment (OAT) for the mother and the fetus exceed the risks [12]. This recommendation was originally based on a few case studies from the 1970s associating opioid withdrawal with fetal death, miscarriage, and preterm labor [13–15].

Retrospective studies have attempted to evaluate the risk of fetal loss with maternal opioid withdrawal. Luty et al. concluded opioid withdrawal in the first trimester may be associated with miscarriage but that it was likely safer in the second and third trimesters [16]. Several studies evaluating opioid withdrawal in pregnancy have not shown significant risk of fetal loss, often with miscarriage rates comparable to population norms [17, 18, 19•, 20]. Unfortunately, these studies are underpowered and contain confounders making the risk of pregnancy loss secondary to opioid withdraw difficult to quantify.

Maternal opioid withdrawal does decrease incidence and duration of neonatal abstinence syndrome [17, 18]. Despite recent studies suggesting fetal safety of medically assisted opioid withdrawal in pregnancy, abstinence of maternal drug use was low and maternal relapse rates range from 59 to 99 % [18, 19•].

We believe opioid withdrawal is an inferior option compared to OAT for pregnant women, and risk of maternal relapse, with inherent overdose mortality risk, outweighs the potential reduction in NAS. In the general population, OAT is the standard of care for opioid use disorder; a standard which should not be altered by pregnancy.

### Treatment Options for Pregnant Women: Opioid Agonist Treatment

OAT is the first-line recommendation for pregnant women with opioid use disorders. The goals of treatment are to manage withdrawal, reduce cravings, and provide opioid blockade (preventing euphoria from illicit use). The goals of OAT in pregnancy are to prevent illicit opioid use which can increase the risk of fetal growth restriction, abruptio placentae, fetal death, preterm labor, and intrauterine passage of meconium. OAT has been shown to increase adherence to prenatal care, reduce illicit drug use, reduce infection exposure secondary to IVDU, such as HIV, HCV, HCB, improve maternal nutrition, and improve infant birth weight [21].

Neonatal abstinence syndrome (NAS) is an expected and treatable condition due to prenatal opioid exposure. Forty to 80 % of exposed neonates develop withdrawal requiring hospitalization and pharmacotherapy. Historically, methadone has been the gold standard OAT choice for pregnant women. Emerging evidence supports the use of buprenorphine as an effective therapeutic option in pregnancy and shows improved neonatal outcomes. A 2010 multicenter randomized clinical trial showed buprenorphine-exposed neonates required, on average, 89 % less morphine to treat NAS, a 43 % shorter hospital stay, and a 58 % shorter duration of medical treatment for NAS compared to methadone-exposed newborns. These results support the use of buprenorphine as a potential first-line medication for pregnant opioid-dependent women [22••].

The advantages of buprenorphine over methadone include a lower risk of overdose, fewer drug interactions, office-based treatment delivery, and shorter NAS course. The disadvantages compared with methadone include potential hepatic dysfunction, lack of long-term data on consequences of fetal exposure for the infant, potential limited efficacy in patients with high opioid debt, requirement of moderate withdrawal symptoms prior to initiation to avoid iatrogenic withdrawal, and an increased risk of diversion (i.e., sharing or sale) [21]. To date, studies have not assessed the impact of maternal opioid addiction severity on newborn outcomes and maternal long-term recovery.

For the provider, the choice of OAT type to initiate must be individualized and is often restricted by availability and access to services. We recommend, whenever possible, to optimize patient autonomy in this decision. Despite buprenorphine’s neonatal advantages, it will not be effective for all women. We suggest the best neonatal outcomes will be achieved by providing the most appropriate and effective treatment for the mother.

## Overdose and the Use of Naloxone in Pregnancy

Currently, opioid overdose is a leading cause of death in Americans 25–45 years of age. Education and expansion of access to naloxone is one evidence-based strategy to address this issue. No study has looked at pregnancy outcomes in patients treated with naloxone for acute opioid overdose, but the use of naloxone for resuscitation in overdose should not be withheld out of concern for the developing fetus. Despite potential negative effects of acute opioid withdrawal on the fetus, including stillbirth, fetal distress, and premature labor [18], prevention of maternal death from opioid overdose is the priority.

## Extended Release Naltrexone Use in Pregnancy

Naltrexone (pregnancy category C) is a non-selective opioid receptor antagonist which has shown promise in treating opioid use disorders by decreasing drug-seeking behaviors, drug cravings, and increasing treatment retention [23–25].

Naltrexone has the potential to treat select opioid-dependent pregnant women while eliminating the risk of neonatal abstinence syndrome; however, limited human data are available on naltrexone’s safety and efficacy in pregnancy. Animal studies using 175 times the recommended human dosing have not shown teratogenic effects [26]. Opioid receptors in fetal mammalian tissue exposed to exogenous opioids or opioid antagonists may alter neurobiology and growth [27]. Studies of human-equivalent dosing of implantable naltrexone in rodents show no changes in offspring’s brain morphometry. Behavioral changes in these naltrexone-exposed offspring suggest possible alterations in morphine receptor sensitivity which may persist [28]. Case reports of depot naltrexone exposure in humans have not shown teratogenic effects [29], and in comparison with MMT, it shows lower PTD rates, higher birth weights, and improved APGAR scores. No significant differences in gestational age at birth or birth weight have been noted between non-exposed human neonates and naltrexone implant exposed [30]. Naltrexone does not demonstrate tolerance mitigating the need for increase doses in pregnancy [31].

Induction of depot naltrexone during pregnancy is a complicated proposal and is not currently recommended. OAT remains the standard of care in pregnancy due to improved adherence to addiction treatment services, prenatal care, and in-hospital delivery [19•]. However, women who are stable on extended release naltrexone who become pregnant, based on human and animal data available to date, could be reasonably continued on naltrexone through pregnancy. Individualized treatment plans weighing the risk of altering therapy (stopping naltrexone) and potential destabilization of maternal recovery must be considered. The risk of relapse and possible overdose with such an alteration in addiction treatment may significantly outweigh the naltrexone exposure risk to the fetus alone. Future research on in utero exposure and perioperative pain management of extended release naltrexone are needed [32•].

## Special Considerations: Trauma, Mood Disorders, Breastfeeding

### Trauma and Substance Use Disorder in Women

Prior traumatic experiences from exposure to physical, sexual, and emotional abuse are common among women with substance use disorder, with estimates ranging from 50 to 80 % [33–35]. Pregnancy can be a vulnerable and triggering time for women with trauma histories. A recent qualitative study by Torchalla et al. of pregnant and newly parenting women with substance use disorder in Vancouver identified that all women reported childhood adversity and 77 % reported prior sexual abuse [36]. This study identified themes of continued adverse experiences persisting into adulthood and high rates of intimate partner violence during pregnancy. Women expressed concern over the possible trans-generational effects of their trauma on their infants, but the majority of women questioned had not received any trauma-specific services, and many expressed ambivalence or reluctance in participating in trauma counseling.

Trauma-informed care, or the principle that service delivery is designed with an understanding of the impact of victimization and trauma, has become more prevalent as a method to care for women in gender-specific substance use treatment programs [37, 38]. Yet, a recent secondary database review from publicly available data sets by Terplan et al. examined 13,000 addiction treatment facilities in the USA and found that trauma-related services were selectively available in urban centers and states with larger populations, and a large percentage of women in treatment programs reported unmet needs [39•]. A case study by Goodman et al. examined the complexities of providing obstetric care for women with comorbid PTSD and substance use and stressed the importance of early screening and a coordinated multidisciplinary approach to improving outcomes for the substance-exposed mother-infant dyad [40].

### Mood Disorders in Women with Opioid Use Disorders

According to the National Survey of Substance Abuse Treatment Services, 45 % of Americans seeking treatment for substance use disorders have co-occurring mental health disorders, specifically depression and anxiety [41].

Infants of untreated, depressed mothers demonstrate reduced scores for motor adaptation and self-regulation, have higher arousal scores, are more difficult to console, and can exhibit developmental delay and poor neonatal attachment [42–44].

Several well-conducted reviews demonstrate the safety of selective serotonin reuptake inhibitors (SSRIs) in pregnancy, with the exception of paroxetine, which may increase the risk of cardiac defects [27]. The most common and the best established adverse effect of SSRIs is poor neonatal adaptation (PNA); PNA is prolonged when SSRIs are combined with benzodiazepines [45]. In addition, SSRIs in combination with opioids have been shown to worsen severity of NAS [46, 47].

The decision of when and how to treat perinatal depression is a complex risk-benefit algorithm. Mild to moderate depression should probably be treated with cognitive behavioral therapy or interpersonal therapy (if available). The studies supporting this are small but CBT and IPT are proven effective treatments for depression [48].

For anxiety disorders, SSRIs should be considered the first-line pharmacotherapy. Benzodiazepines are relatively contraindicated in women with opioid use disorders. For patients on OAT, benzodiazepines exacerbate the sedating effect of methadone and increase accidental injury, especially in women prescribed with buprenorphine [49]. Neonates exposed in utero to both OAT and benzodiazepines experience prolonged NAS [46]. The authors caution that benzodiazepine use disorder and benzodiazepine diversion are growing public health concerns and recommend vigilance in prescribing practice.

### Breastfeeding and OAT in Pregnancy

Breastfeeding is recommended for women on OAT. Methadone and buprenorphine are both lactation category C and transferred via the breast milk but in amounts <1 % of the maternal dose [50–53]. Published guidelines from the American Academy of Pediatrics (AAP), the American College of Obstetricians and Gynecologists (ACOG), and the Academy of Breastfeeding Medicine (ABM) all support breastfeeding for women stabilized on OAT [54–56]. The 2015 ABM statement includes criteria for encouraging breastfeeding among mothers with the following: (1) compliance with a substance use disorder treatment program, (2) consistent prenatal care, (3) negative urine toxicology screen at the time of delivery, and (4) negative urine screens for 30–90 days prior to delivery [54]. One institution expanded on the ABM criteria by (1) defining consistent prenatal care as attendance of at least 50 % of scheduled visits, including two visits within the last 2 months, and (2) creating a “4-week guideline” of no positive urine drug screens for breastfeeding initiation, leading to subsequent improved breastfeeding rates [57]. The use of prenatal urine drug screens as a predictor for postpartum recovery remains unknown despite these recommendations.

For women on OAT, breastfeeding can be a complex issue. Despite AAP, ACOG, and ABM statements endorsing the benefits of breastfeeding on OAT and its safety with coexisting hepatitis C status, policies differ among institutions. Policies may reflect provider fear of maternal relapse, concern for concurrent psychiatric medications, and how these may complicate the course of NAS [58–60]. However, numerous studies have shown a strong association between breastfeeding and improved NAS symptoms, with reduction in need for pharmacotherapy and shortened hospitalizations [61–64]. In addition, there are significant maternal benefits including reinforcing sobriety, enhancing maternal self-esteem, and the promoting mother-infant bonding. Previous studies have not differentiated between direct breastfeeding and pumped breast milk, nor mixed feeding versus exclusive breastfeeding. The benefit is likely related to maternal presence at the bedside, skin-to-skin contact, and engagement in care.

## Conclusion

Over the past 10 years, there has been a surge of illicit opioid use in the USA. Pregnant women with opioid use disorder are at an increased risk for negative birth outcomes. Limited access to care, social stigma, inadequate insurance coverage, and fear of legal consequences are some of the many barriers to care for pregnant women. The current standard of care for opioid use disorder in pregnancy is an opioid agonist treatment. Assisted opioid withdrawal is not recommended due to the high risk of relapse. Extended release naltrexone is a viable option for carefully selected women. With further study, the use of buprenorphine + naloxone (dual-therapy) may replace treatment with buprenorphine (mono-therapy) in pregnancy. Opioid use disorder treatment should be gender specific, and prenatal care programs should incorporate trauma-informed care and provide mental health services. Initiation of pharmacologic treatment for mental health disorders must consider maternal efficacy, maternal and fetal safety, and risk of NAS potentiation. Breastfeeding for women on OAT is safe and should be supported to decrease onset, duration, and severity of NAS.

In our current healthcare system, the expansion of safe, effective, and dignified multidisciplinary treatment options for pregnant women with opioid use disorders is greatly needed. Expansion of access to care for pregnant women can be achieved by increasing the number of buprenorphine-waivered obstetric providers and expanding comprehensive obstetric and addiction medicine models of care. Obstetric providers must strive to decrease the stigma associated with the disease, reduce the barriers to care, and implement comprehensive programs to meet the rising demand.

Our recommendations for health care systems to provide to pregnant women with opioid use disorder can be found in Table 1.

**Table 1:** Recommendations for health care systems to provide to pregnant women with opioid use disorder

o *Access to opioid agonist treatment options* Methadone or buprenorphine o *Access to obstetric care* Recovery-affirming and trauma-informed Comprehensive obstetric and addiction medicine services Group prenatal care as an option o *Access to psychiatry consultation*: assessment and treatment options for co-occurring disorders o *Access to behavioral health counseling*: weekly individual or group counseling o *Resource guides for community-based relapse prevention* Mutual aid support groups Mothers-in-recovery groups o *Development of enhanced postpartum care*: program development to intensify recovery support potentially utilizing peer supports Close follow-up (<2 weeks from delivery) Allow for multiple postpartum visits Consider visits every 2 weeks for 3–6 visits Breastfeeding/lactation support Screening/treatment for postpartum depression Transition to a primary care provider familiar with opioid use disorder and its treatment	

## Footnotes

1 Due to opposition from health care professionals and patient advocacy groups, this law is currently being repealed.
